# Bibliographical Notices

**Published:** 1855-05

**Authors:** 


### BIBLIOGRAPHICAL NOTICES.

*Report of the Sanitary Commission on the Epidemic Yellow Fever of* 1853*; published by authority of the City Council of New Orleans.* By E. H. BARTON M. D., and by Drs. AXTON, MCNEILL, RIDDELL and SIMONDS. New Orleans, 1854, pp. 542.

If the calamitous invasion of the pestilence of 1853, had produced no better ultimate effect upon the sanitary fortunes of the Crescent City than the development of this voluminous and most elaborate report, there would be reason for material consolation in the vitally important lesson it thus teaches for the future in regard to the etiology and prophylaxis, or rather aggravation and modification, of the dreaded epidemic.

We have been greatly interested in the copious details and various practical suggestions of Dr. Barton and his colleagues, and can safely recommend their manifesto to the attention of all who take interest in the study of public hygiene, as well as to all investigators of the course of the present epidemic scourges of the world. The growing extent and popularity of these sanitary inquests afford gratifying evidence of a far more enlightened appreciation of the benefit of hygienic regulations than formerly prevailed in the councils of the nation and among the people generally; and we are so fully convinced of the value of the feeling thus awakened, that we are anxious to encourage, in every proper way, a work that is so well calculated to make a good impression as the one before us. There is much striking evidence collected in its pages; and the practical conclusions are so freely and forcibly presented, notwithstanding a little very natural extravagance, that we earnestly hope it may exert a lasting influence, not only upon the community to whom it was addressed, but upon their more favored neighbors in other portions of this continent. In fact much that is stigmatized and recommended in relation to yellow fever in the South will equally well apply to the cholera and other malignant diseases in any portion of the country, and may hence be profitably pondered over in all quarters of the land.

We have no space or time to spend upon a regular review of the series of reports and other documents contained within the volume. A brief account of some portions of its matter must answer our purpose of introducing their general scope and meaning to the reader.

A sanitary commission appointed by the authorities of New Orleans was directed—

"1st.—To inquire into the origin and mode of transmission or propagation of the late epidemic yellow fever.

"2d.—To inquire into the subject of sewerage and common drains, their adaptability to the situation of our city, and their influence on health.

"3d.—To inquire into the subject of quarantine, its uses and applicability here, and its influence in protecting the city from epidemic and contagious maladies, and

"4th.—To make a thorough examination into the sanitary condition of the city, into all causes influencing it, in present and previous years, and to suggest the requisite sanitary measures to remove or prevent them, and into the causes of yellow fever in ports and other localities having intercourse with New Orleans.

"The Commission immediately organized and proceeded with due diligence to the fulfilment of the important task confided to it. It issued circulars embracing all the points for examination, and distributed them among the medical faculty and citizens here and the adjoining States, and to every quarter of the yellow fever region, whence information could be expected to enlighten its judgment on the subjects to be considered. It sat as a Court of Inquiry in this city daily for about three months, eliciting and inviting information from every accessible source.

"When this field had been sufficiently explored, it deputed its various members to visit different parts of this and the adjoining States where the epidemic had existed, to institute inquiries upon like matters and report upon them. One member was sent to visit the various Eastern cities, to obtain information of their sanitary condition, ordinances and usages. He was likewise instructed to visit Washington, to apply to the Government of the United States for aid in obtaining through our Diplomatic and Consular agencies throughout the yellow fever zone, whatever information our circulars called for, or that would advance the cause we were engaged in."

The result of the extended investigation, thus provided for, appears in a large amount of printed testimony furnished by a considerable number of intelligent medical practitioners and other observers in different places; several series of valuable maps and charts and meteorological tables prepared by Dr. Barton; a special report by Drs. Axton and McNeill on the first subject of inquiry; another by Dr. J. L. Riddell on the second subject; another by Dr. J. O. Simonds on the third; and lastly, a general report on the whole subject by the chairman, Dr. Barton, under the fourth head of the category given.

The first subsidiary report is well written and clearly drawn up. The conclusions are, that the fever had a local origin and was not contagious.

The report of Dr. Riddell is a capital one in every respect. Brief, clear and comprehensive, elegant in style and arrangement, and accompanied with engraved illustrations, it strikes us as especially useful in its suggestions for the immediate and thorough cleansing and drainage of the city.

The last of these minor reports—that upon the quarantine by Dr. Simonds—although well arranged and written, is not as full and perhaps not as conclusive as it might have been, had not the unfortunate impairment of his vision prevented him from engaging in the more extended reading and examination of the testimony indispensable to a full discussion of so embarassing a question. He would seem to be behind the age in adhering to the quarantine—at least the committee, for they are unanimous, are in this matter not in accordance with the the tendency of British and French experience and with a prevalent feeling in America against the quarantine as usually enforced. We are inclined to agree with the reporter in this view, although we cannot stop to argue in its favor. There can be hardly any dispute as to the propriety of the exclusion of any vessel suspected of impurity during the season of epidemic liability; and at all events, if there be a reasonable doubt in regard to the safety of admitting what may become the origin or source of mischief, it is no more than fair to give the inhabitants of each city the benefit of that doubt. The real inquiry seems to us to concern rather the nature and extent of the quarantine in each case, than the quarantine itself. The only question in which we discover any difference of opinion among the members of the committee, is that of the local origin of the fever. Dr. Barton asserts the domestic origin to have been incontestably established, but Drs. Riddell and Simonds deny the certainty alleged by Dr. Barton, and prefer remaining, like many other equally respectable authorities, unsettled in their views.

The report of Dr. Barton, with its accompanying maps, charts, hygrometrical and other meteorological tables, occupies, as it should, by far the lion's share of the whole production. It is a monument of painstaking industry, abounding in zealous discussion and explanation of his ideas respecting the origin and causes of yellow fever and the best mode of counteracting them. The two grand principles, the exposition of which he values above all others in his confession of sanitary faith, we may give in his own words:

"The one is that yellow fever is and always has been, here and elsewhere, a *preventable disease*, and

"The other is, that the presence of two general hygienic conditions are absolutely indispensable to the origination and transmission of the disease—the one of them, atmospheric—the other terrene. These must meet *in combination*, or there will be no result. The absence of *one*, as to *this* disease, is as the absence of *both*, and as one of these conditions is almost wholly within the control of man and the other partially so, it must follow that his power extends to its prevention and expulsion.

"These two principles constitute the bases of all the preventive and remedial measures with which the Report closes, and which were specially devised for practical execution through the ministrations of the city authorities."

We would be glad to be able to notice, to some extent at least, the table in which he has presented what he calls the "Climatic or meteorological elements of Cholera and Yellow Fever at New Orleans" in different years. The deductions from this table are among the most interesting peculiarities of the whole report. Although they may afford no decisive information with the present data, they are certainly very hopeful indications of an extensive and yet unexplored field.

The conclusions of Dr. Barton are substantially the same as those of the British General Board of Health, which he quotes in full, with the strongest expression of approval. We could not, if it were desirable, repeat those conclusions here, or examine them in detail. Suffice it to say that yellow fever is local in its origin; that the conditions which influence its localization are known, definite, to a great extent removable, and very much the same as those of cholera, and all other epidemic diseases; that it becomes more rare, more mild, or disappears, in proportion as the local causes are abated or removed, and that, consequently, the means of protection are not quarantine restrictions and cordons, but *sanitary works and operations*, having for their objects the removal of the sources of disease, or the removal of the population from exposure within the infected districts to the operation of those sources.

No resident of New Orleans, or indeed of any other city of our Union, could candidly examine the exposition of Dr. Barton without instruction; and we do hope, for the sake of the common weal, that it may be carefully and widely studied, in the North as well as in the South. There are many hints to be found in it which would be well worth attending to in places north of Mason & Dixon's line, no less than in the warmer regions of our more exposed neighbors.

It was intended for the public at large, and is therefore addressed to the nation instead of the profession; and should it meet with but a small share of the consideration to which it is entitled, the people of New Orleans cannot fail sooner or later to derive a lasting and inestimable benefit.

Strongly impressed with this feeling as, notwithstanding the rather discouraging bulk of the report, an attentive perusal of Dr. Barton's representations has rendered us, we find with great regret that the Reporter has encountered the most bitter and unreasonable opposition, in the pages of a New Orleans medical journal, and from a fellow practitioner—the very last source from which such an offering of professional beneficence ought ever to have been assailed. The whole spirit, style, and tendency of this home-made critique are such as to deprive it of any power, except a reactionary one in favor of the object of attack. We lament its appearance, however, as creditable to the profession of the country, and especially of the afflicted city itself, in which so much has been done, both at the risk and sacrifice of life and health, by the members of that profession, in support of a well earned reputation for devotion to the best interests of science and humanity. It is a great pity that any one occupying the responsible position of a journalist should allow his private animosity so far to get the better of his sense of propriety and justice, as to betray him into the gratuitous personal abuse of an unoffending scientific neighbor, and, through this neighbor, of the municipal authorities under whom he was called upon to engage in a public duty of the highest importance and respectability.

*On the Construction, Organization and General Arrangements of the Hospitals for the Insane.* By THOMAS S. KIRKBRIDE, M.D.

*Report of the Pennsylvania Hospital for the Insane, for the year* 1854. By THOMAS S. KIRKBRIDE, M.D.

*Annual Report of the Trustees and Superintendents of the State Lunatic Hospital of Pennsylvania, for* 1854.

We would be sorry should our interest in the subject treated of in the works which we prefix, be estimated by the length of our remarks. In truth, there is no topic on which we would more willingly dwell; but time and space permit us only to say that "Kirkbride on Hospitals for the Insane" exhausts the theme, and both reports are replete with instructive information on the nature and treatment of Insanity.

